# Epidemiology of cervical cancer in Latin America

**DOI:** 10.3332/ecancer.2015.577

**Published:** 2015-10-08

**Authors:** Luis G Capote Negrin

**Affiliations:** Clínica Luis Razetti, Consultorio 65, Av Este con Calle Sur 21, Candelaria, Caracas, 1010, Venezuela

**Keywords:** cervical cancer, Latin America, human papillovirus (HPV)

## Abstract

The basic aspects of the descriptive epidemiology of cervical cancer in Latin America are presented. A decrease in the incidence and mortality rates has been observed in the period from 2000 to 2012 in all countries across the region, this has not occurred at the same proportions, and in many countries, observed figures of incidence and mortality are among the highest levels in the world. In Latin America, calculating a mean measure of the numbers from the GLOBOCAN data from 2000 to 2012, we can observe a difference of up to fivefold of the incidence (Puerto Rico 9,73 Vs Bolivia 50,73) and almost seven times for mortality (Puerto Rico 3,3 Vs Nicaragua 21,67).

A report of the epidemiology, risk factors, and evaluation of screening procedures regarding the possible impact of the human papillomavirus (HPV) vaccine I in the prevention of cervical cancer is presented.

## Introduction

Cervical cancer remains the number one cause of mortality due to malignant neoplasm among 20 to 40-year-old women in Latin America, and the third most common cause of cancer death in females, second only to breast cancer and lung cancer.

In a recent study, the World Health Organisation (WHO) admitted that as many as 529,409 women worldwide will be diagnosed with cervical cancer, and 274,883 will die from the disease. No less than 80% of this burden is borne in less developed countries [1].

In America, it is estimated that there are around 92,136 cases and 37,640 deaths per year, representing a significant economic loss, projected at being potentially greater than US$ 3,6 billion [2].

This is a pathology that has been recognised for more than 50 years as being a disease linked to the sexual habits of women or their partners [3, 4, 5] and furthermore whose incidence clearly disproportionately impacts women from the poorest social strata, and the most economically disadvantaged regions [6]; to the extent that mortality rates from this cancer are three times higher in Latin America and the Caribbean than in the United States and Canada [2].

The variations between countries in the region, for the period from 2000 to 2012, taken from the GLOBOCAN reports [7], show a difference in the rates, standardised per 100,000 women, of up to five times the incidence (Puerto Rico 9.73 v Bolivia 50.73) and almost seven times the mortality (Puerto Rico 3.3 v Nicaragua 21.67). These differences are more strongly linked to unequal access to control measures than as a consequence of differences in the sexual conduct of the populations.

## Descriptive aspects of cervical cancer in Latin America

**Figure 1.**
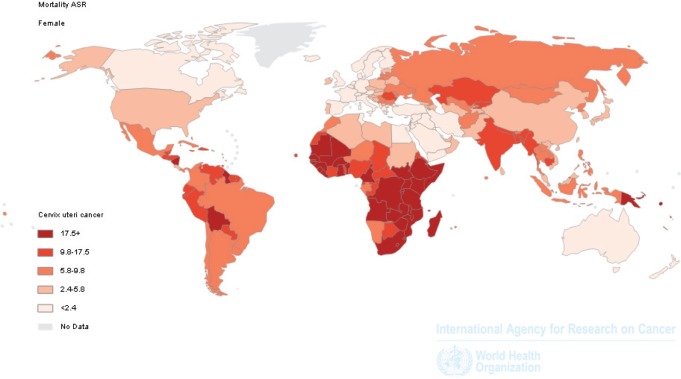
Cervical cancer around the world. Estimated mortality rates.

**Source:** GLOBOCAN 2012 (IARC).

**Figure 2.**
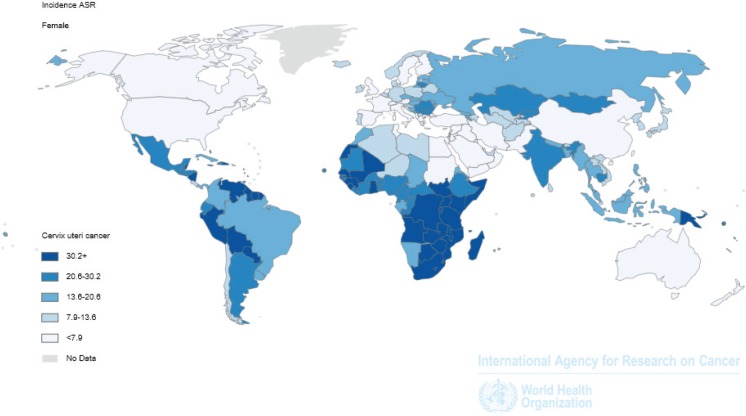
Cervical cancer around the world. Estimated incidence rates.

**Source:** GLOBOCAN 2012 (IARC).

It is useful to draw comparisons with data on the global incidence of cervical cancer. Figures 1 and 2 show the specific distribution, which has led to the development of cervical cancer known as third most common cancer worldwide. Economically better off regions clearly show lower rates, with notable exceptions in North Africa, the Arabian Peninsula, and some near Asian areas, which while they are not part of the most economically developed areas of the world, show low rates, possibly due to the up keep of religious mores favouring conservative sexual conduct; a similar observation was presented by Drain *et al* in a work analysing determinants of cervical cancer rates in underdeveloped countries [8].

In Latin America, cervical cancer puts an enormous strain on the health system [2], presenting as the third most common cause of female cancer death, second only to lung and breast cancer; although in some countries, such as Honduras, Nicaragua, El Salvador, Bolivia, Paraguay, and Ecuador, it remains the principal cause of female cancer deaths. Nonetheless, it is one of the most preventable and curable forms of cancer, as shown by cancer statistics from developed countries.

In Latin America for 2012, almost 70,000 cases and 28,000 deaths from cervical cancer occurred, according to information compiled from GLOBOCAN data [7].

The Pan American Health Organisation estimate for 2012 was around 36,000 deaths from this type of cancer across America with 80% of these deaths relating to Latin America and the Caribbean www.paho.org/cancer/cervicalcancer [9].

The descriptive epidemiological method is a valuable tool when referring to the social behaviour of disease, especially in relation to chronological trends, geographical distribution or individual factors.

This article is limited to those basically descriptive aspects of the epidemiology of cervical cancer in Latin America, as well as to understand its natural history and the evaluation of screening procedures and the possible impact of the vaccine against human papillomavirus (HPV), as a contribution to the understanding and control of the disease.

Evaluation of chronological trends and geographical distribution of cervical cancer, observed between 2000 and 2012, shows a reduction in mortality in absolute terms, from 31,000 deaths in 2000 to 28,000 in 2012 [7]. This trend is obviously not proportionally distributed, and while in the majority of countries, there has been a reduction in mortality, but mortality rate increases in some countries.

To better appreciate the real variation in risk, we measure it using one indicator, such as the standardised rate, which takes into account the population structure by age group and as a benchmark, the adjusted Segi world standard population.

**Figure 3.**
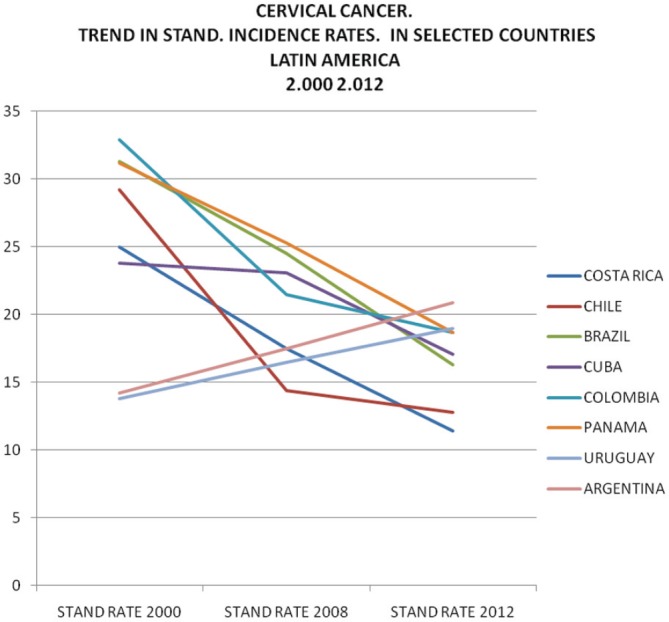
Cervical cancer. Standard rates. Incidence selected countries in Latin America 2000–2012.

**Source:** GLOBOCAN, 2.000, 2.008, 2012.

**Figure 4.**
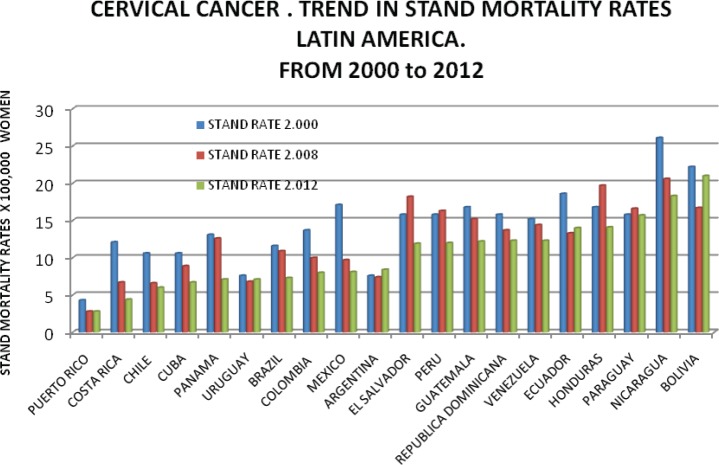
Cervical cancer trend in standard mortality rate from 2000 to 2012 in Latin America. Standard mortality rates X 100,000 women.

**Source:** GLOBOCAN 2000, 2008, 2012.

**Figure 5.**
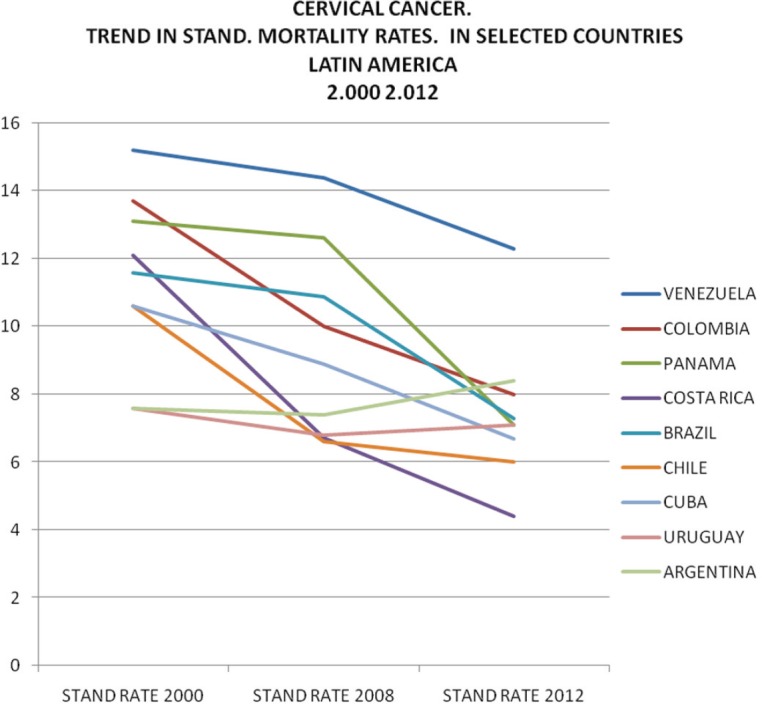
Cervical cancer. Trend in standard rates. Mortality selected countries in Latin America 2000–2012.

**Source:** GLOBOCAN 2000, 2008, 2012.

Tables 1 and 2 and Figures 3, 4, and 5 show trends in the incidence and mortality by country for the given period from 2000 to 2012, corresponding to cuts in 2000, 2008, and 2012; taken from GLOBOCAN data [7]. Table 2, which relates to mortality, shows that rates in the majority of countries fell over the analysed period, with the exception of Bolivia, which showed a significant increase, Ecuador with a slight increase and Argentina and Uruguay while they have a low incidence showed a small increase in 2012, as can be seen in Figures 4 and 5.

The incidence of cervical cancer in Latin America shows a tendency over time very similar to mortality; a marked variation can be seen between countries in Table 1 and Figure 3.

Individual factors include those variables linked to the personal characteristics of the women affected by the problem being studied. More than five decades of epidemiological evaluation has indisputably determined its relationship to the sexual habits of women and their partners; [4] as well as other characteristics, which constitute the risk profile associated with the greater probability of this cancer presenting in certain women [3].

A descriptive evaluation of the magnitude of the problem and its principal defining characteristics is shown below:

### Magnitude of the problem

Along with the data mentioned in previous paragraphs and supported by tables, quantifying the significant scale of the problem by incidence and mortality figures, it would be useful to use a different social and economic disease indicator. Over the past few decades, years of potential life lost have been used for this. However, their calculation requires access to information on the age distribution of women with cervical cancer across all Latin American countries, which is fairly difficult to obtain.

Other essential statistics are those relating to life expectancy among the female population in the countries analysed, which can be obtained from World Bank indicator reports [10].

**Figure 6.**
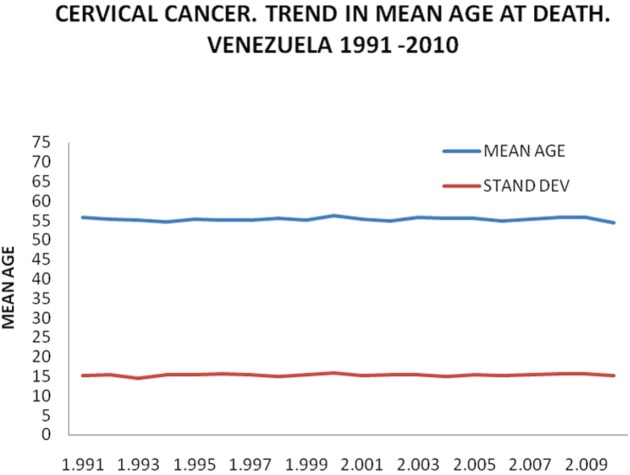
Cervical cancer. Trend in mean age at death. Venezuela 1991–2010. Mean age.

**Source:** Central cancer. registry. mortality annuary. mpps. venezuela.

**Figure 7.**
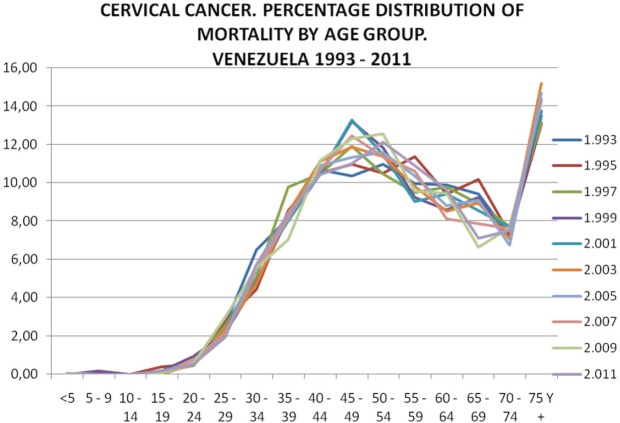
Cervical cancer. Percentage distribution of mortality by age group 1993–2010.

**Source:** Central cancer. registry. annuaries of mortality. mpps. venezuela.

**Figure 8.**
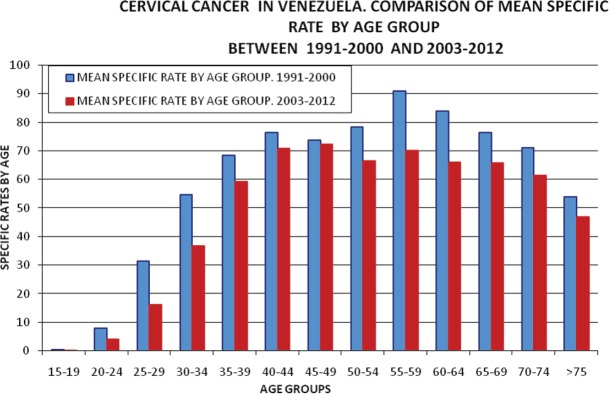
Cervical cancer in Venezuela. Comparison of specific rates by mean ages 1991–2000 versus 2001–2010. Specific rates by age groups.

**Source:** Central cancer. registry. oncology program. mpps. venezuela.

To avoid the stated difficulties, a mathematical model was applied based on the assessment carried out over the course of more than 20 years monitoring the behaviour of cervical cancer in Venezuela. This has shown a marked stability in indefinitely maintaining an average age of death of 55 years and a fairly stable percentage distribution by age group, as is shown in Figures 6, 7, and 8.

This allowed us to maintain the hypothesis, to be set out in a subsequent study, of similar behaviour in all other Latin American countries.

By applying the same percentage distribution by age at death for each country, it is assumed that the average age of death will equally be very close to 55 years. In our experience of analysing trends of mortality due to cervical cancer over a number of years, we have noticed that the average age is very close to the median in each annual series, which moreover have a pattern that approximates to a normal curve; consequently, taking the value of the average age at death and subtracting it from life expectancy would give us a value close to the average number of years of life lost by each death, and by multiplying this by the total number of deaths per period, we can estimate somewhat approximately the real number of years of life potentially lost, as shown in Table 3, which establishes for the region a loss of 633,000 potential years of life, especially in areas where the woman is the fundamental factor in a nuclear family.

Knowledge of distribution by age group is determined by the design of control strategies, meaning that its constant monitoring is needed for detecting possible variations over time.

As previously stated, in Venezuela, monitoring has been maintained, based on incidence rates by age group, expressed in classes spanning 5 years, which we have maintained since 1980.

Very similar curves have been observed, rising rapidly from class 20–24, up to 45–49, with the ascent slowing up to 55–59; beyond this point, the incidence rate begins to decrease.

Figure 8 shows a comparison between the decades 1991–2000 and 2001–2006, during which periods a moderate decrease was seen in the rate of incidence, equally reflected across all age groups.

Therefore, this renders questionable the frequent affirmation by clinical specialists pointing to an increase in cases in younger women, which is simply an effect of population (larger current population in this age group means more cases, but not an increased risk, and still less a change in the natural history of this pathology).

As we have already shown, the average age of the cases and of death has remained stable in Venezuela (Figure 6) and there is a substantial enough basis for the stability of the natural history of this cancer [11] to suppose that the same must be happening in the rest of Latin America.

Demographic factors include illiteracy, poverty and poor hygiene practices, little access to health services or attention to investigatory programmes based on periodical smear testing. These factors are consistently linked to higher incidences of, and mortality due to, cervical cancer, from which comes the connotation that it is a third world cancer, or the view that a high mortality rate from cervical cancer is an indicator of underdevelopment, but it is certain that the impacts of these factors are given in determining the influence of risky sexual habits and the lack of timely and appropriate access to prevent and control measures.

Other factors, such as smoking habits, are indicated in various studies to be tied to an increased risk of cervical cancer, linked to a doubling of risk to women smokers and correlating positively to the greater intensity and duration of the habit [12]. The discovery of nicotine and the derivatives of tobacco smoke in cervical mucus, signalled by Schiffman, [13] suggest a possible biological mechanism through immune-suppression, which encourages infection by the HPV.

Nutritional deficiency is a risk factor which has been closely evaluated, especially in relation to its potential application in chemoprevention. Evidence based on epidemiological studies exists showing that nutritional deficiency, in particular in vitamins A and C, lead to increased risk of cervical cancer. In one International Agency for Research on Cancer (IARC) study, high- and low-risk regions are compared, and a decreased risk is shown in areas with increased consumption of vitamin C and beta carotene [14]. Other studies in Italy and the USA support these results [15].

A multicentre study of cases and controls did not find any relationship between increased risk and levels of carotenoids, vitamin A or vitamin C, measured through information on the consumption of 75 types of foods [16].

Over the course of many years, cervical cancer has been linked with sexual and reproductive factors. The first antecedents date from the observations of Doménico Rigoni-Stern in 1842 [17] who described his discoveries in a statistical investigation across a number of decades of cancer mortality in the population of the Verona region of Italy, which showed the absence of cervical cancer in nuns, and a greater incidence in married or widowed women than among single women; this was followed almost a century later by the publication of similar discoveries by Lebian Gagnon [18] in Quebec.

However, from the 1960 onwards, numerous works were published, many of which were included in the systematic revision by I D Rotkin [19] in 1973, aiming to identify an infectious agent which, by transmission through sexual contact, could be the cause of this type of cancer.

It was Harald Zur Hausen [20] who in 1983 identified the DNA of the HPV in the cancer cells in the cervix and subsequently with the epidemiological work of Nubia Muñoz, Xavier Bosh and Cols, [21] the hypothesis was confirmed, that is, that the persistent infection with types 16, 18, 58, 31, etc. of the HPV, constituted the necessary factor, although not sufficient for the development of this malignancy.

Acceptance of HPV as a necessary factor in the development of cervical cancer lends value to the description of other sexual factors that could play a complementary role; these include initiation of sexual activity at a young age, which could be characterised as being under 18 years old, and risky sexual habits, defined as having more than two partners over the course of a sexually active life, with whom the relationship lasted not less than three months, or similar sexual habits in the male partner.

The increased risk posed by initiation of sexual activity at a young age is attributable to immunological immaturity of the cervical epithelium which allows for a viral infection and not as a factor in itself.

Risky sexual habits are directly linked to the possibility of infection by one or a number of the high-risk forms of HPV. The particular distribution of cervical cancer in certain regions of the world, which show the very low levels of mortality associated with restrictive sexual habits, consistently reinforces the role of sexual factors.

New Guinea is one of the examples that can support this hypothesis. It is an island located to the north of Australia, whose western portion belongs to Indonesia, and hence, the majority of the population are practising Muslims and mortality rates are much lower than the eastern part, Papua New Guinea (Figure 1), an independent country where only a minority of the population belong to this religion; thus highlighting variations in sexual habits as the cause of the marked difference.

The natural history of cervical cancer has been recognised in the last four to five decades, without there have been major changes beyond differences in the nomenclature of precancerous lesions or the identification of the HPV as the necessary agent, but not sufficiently to have developed and evolved into a malignant neoplasm. The importance of understanding the natural history of this pathology is in its application to control measures. As can be shown in Table 4, pre-cancerous lesions can be detected 5 to 10 years before women become clinically symptomatic [11], thus identifying these lesions gives a good chance of arresting the development of the pathology.

Early detection monitoring procedures for cervical cancer or its precursor lesions have been in place for some time, ever since the early development of the Aurel Babes and George Papanicolou cervicovaginal cytology technique [22] and colposcopy developed by Hans Heinselman [23].

Cervicovaginal cytology has had a significant impact on the control of this disease, despite having numerous detractors. However, it is not a simple method, as in order to be effective, it requires a well-structured organisation, supported by a population-based programme and a health system with well-defined levels, with complete decentralisation of the regular collection of cytology samples at clearly defined intervals, and with a protocol based on the epidemiological knowledge and potential of the cytology method. It requires well-equipped cytopathology laboratories, capable of processing no fewer than 40,000 smears annually, with strict quality controls and sufficiently qualified staff; and it also needs clinics for referring cases (Cervical Pathology Clinic), located strategically and with trained staff, able to promptly assess and treat (if necessary) all cases identified by cytological testing, who must have been located, given an appointment and treated appropriately, according to the programme protocol.

It is evident from the above that it is not a simple method, and it is also clear that it can operate with good results on an “opportunistic” basis, as in the USA, but this is only possible in countries with a high income per inhabitant, with costs that are multiples of those of scheduled, population-based research, as has been demonstrated in Scandinavia, Great Britain and many regions of France, Italy or Germany [24].

Appropriate, regular, well-structured access to cytology has been the main factor in determining the difference between countries at high risk and those at intermediate or lower risk (Table 5). The knowledge, though limited, obtained from Pan American Health Organisation (PAHO) reports [25, 26] on the percentage of cytology cover in Latin American countries fits fairly precisely inside the epidemiological profile of countries at low risk with mortality rates below 10, countries at intermediate risk with rates of 10 to 15, and countries at high risk, above 15. Some countries do not report on their cytology cover, such as Puerto Rico, but this should be close to the average for the USA which is 80%. Others, such as Venezuela or Panamá, report only cover corresponding to the Ministry of Health, and the percentage should therefore be around 50%. This correlation, which provides a fairly good match between good rates of cytology cover and low mortality rates and vice versa, is why we have accepted that the most decisive risk factor in mortality due to cervical cancer is the absence of appropriate cytological testing.

Other detection methods, such as direct visualisation of the cervix with acetic acid (visual inspection with acetic acid: VIAA) or with iodine (visual inspection with Lugol’s iodine: VILI) carried out by nurses or trained technicians, have gained importance in recent years, and these may be an option in rural populations with an under-developed health system [27]. With the advent of molecular biology techniques applied clinically for the diagnosis of high-risk types of HPV, which offer promising prospects in the more efficient detection of high-grade intraepithelial lesions [28], and which are gaining ground as costs are falling, and which offer a greater potential for large-scale use, we will thus have tools that facilitate monitoring and reduce the huge health and socioeconomic burden represented by cervical cancer in Latin America.

## Vaccines against HPV

With the approval in 2006 of two vaccines against the high-risk HPV types 16 and 18 (which are present in 70% of carcinomas of the cervix) and their potential to reach types 31, 33, and 45 by cross immunity (which would provide approximately 10% additional protection), a powerful weapon is available for preventing approximately 85% of the risk of contracting cervical cancer in the coming years [29].

The majority of Latin American countries have begun HPV vaccination in recent years, but there is insufficient information available on the rates of cover achieved. Although appropriate vaccination levels have been reached, the impact of the effectiveness of the vaccine in preventing cervical cancer will not be observed for about 20 years. Screening procedures with the methods and adaptations specific to each region should therefore be maintained, along with efficient vaccination cover, to achieve effective control of cervical cancer in coming decades.

**Figure 9.**
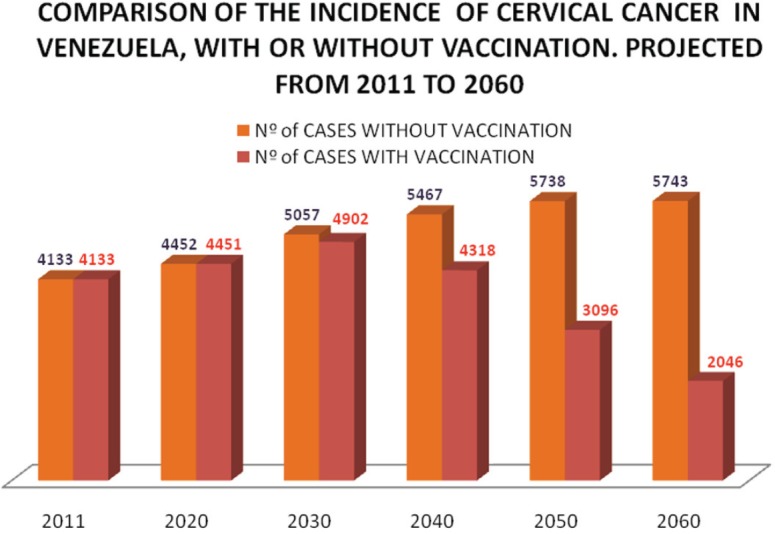
Comparison of the incidence of cervical cancer in Venezuela, with or without vaccination projected from 2011 to 2060. Number of cases without vaccination. Number of cases with vaccination.

**Source:** Central Cancer Registry, Oncology Programme, MPP Health.

**Note:** 85% Efficacy is attributed to the vaccine.

**Figure 10.**
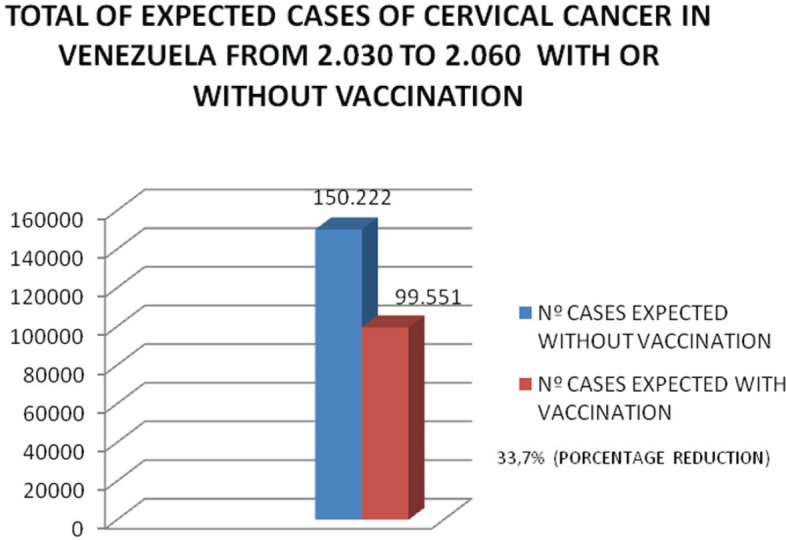
Total expected cases of uterine cancer in Venezuela from 2030 to 2060 with or without vaccination. Number of cases without vaccination. Number of cases with vaccination. 33.7% reduction.

**Source:** Central Cancer Registry, Oncology Programme, MPP Health.

**Note:** 85% Efficacy is attributed to the vaccine.

In an empirical mathematical model, which we use to measure the long-term impact of vaccination in Venezuela and on the premise that the current mortality rate remains stable or shows a slight downward trend, results would begin to be observed from the next 15 years onwards and the greatest impact would be achieved in about 30 years, as shown in Figures 9 and 10, which show a reduction of approximately 50,000 cases between 2030 and 2060, with a percentage reduction of 30.7%; a rapid fall in the number of cases should occur from 2060.

## Conclusion

Cervical cancer has affected the human race for millennia and has wreaked havoc owing to its social and economic scale and impact, particularly in the disadvantaged regions of the world. The fight to control it has been slow; however, it is a clear example of how the development of medical knowledge in diagnosis, therapy, and prevention is paying off in terms of health.

The advent of vaccines to prevent infection by HPV types 16 and 18, which in worldwide studies have proved to be linked to 70% of the risk of suffering invasive cervical cancer and appear to have cross immunity for types 31, 33, and 45, as well as new research prospects involving low-cost procedures and potential widespread application for the detection of HPV risk types, suggest that the fight against cervical cancer is on the way to being won and that this achievement will undoubtedly have a key role in epidemiology.

## Figures and Tables

**Table 1.** Incidence of cervical cancer in America 2000, 2008, and 2012.

Countries	Incidence 2000		Incidence 2008		Incidence 2012		Mean Standard Rate 2000–2012
	Number	Standard Rate	Number	Standard Rate	Number	Standard Rate	
Argentina	2953	14.2	3996	17.5	4956	20.9	17.53
Bolivia	1807	58.1	1442	46.4	2029	47.7	50.73
Brazil	24445	31.3	24562	24.5	18503	16.3	24.03
Chile	2321	29.2	1478	14.4	1441	12.8	18.80
Colombia	5901	32.9	4176	21.5	4661	18.7	24.37
Costa Rica	424	25	403	17.5	297	11.4	17.97
Cuba	1586	23.8	1603	23.1	1287	17.1	21.33
Ecuador	2231	44.2	1666	27.1	2094	29.0	33.43
El Salvador	1041	40.6	1145	37.2	823	24.8	34.20
Guatemala	1432	39.6	1530	30.5	1393	22.3	30.80
Honduras	833	39.6	1014	37.8	991	29.4	35.60
Jamaica	489	43.4	624	45.7	392	26.3	38.47
Mexico	16448	40.5	10186	19.2	13960	23.3	27.67
Nicaragua	997	61.1	869	39.9	934	36.2	45.73
Panama	389	31.2	426	25.3	351	18.7	25.07
Paraguay	768	41.1	864	35	1022	34.2	36.77
Peru	4101	39.9	3445	34.6	4636	32.7	35.73
Puerto Rico	252	10.3	209	7.5	259	11.4	9.73
Dominican Republic	1290	38.4	1299	29.7	1507	30.7	32.93
Uruguay	307	13.8	348	16.5	402	19.0	16.43
Venezuela	3904	38.3	4116	31.4	4973	32.8	34.17

Standardised rates per 100,000 women according to the world population model

**Source:** GLOBOCAN (2000, 2008, 2012).

**Table 2.** Mortality due to cervical cancer Latin America (2000, 2008, 2012).

Countries	Mortality 2000		Mortality 2008		Mortality 2012	
	Number	Standard Rate	Number	Standard Rate	Number	Standard Rate
Argentina	1585	7.6	1809	7.4	2127	8.4
Bolivia	661	22.2	638	16.7	845	21.0
Brazil	8815	11.6	11055	10.9	8414	7.3
Chile	860	10.6	721	6.6	734	6.0
Colombia	2339	13.7	2154	10	1986	8.0
Costa Rica	197	12.1	158	6.7	116	4.4
Cuba	730	10.6	684	8.9	569	6.7
Ecuador	892	18.6	832	13.3	1,026	14.0
El Salvador	387	15.8	563	18.2	388	11.9
Guatemala	566	16.8	717	5.2	672	12.2
Haiti	1326	53.5	353	10.1	575	14.6
Honduras	329	16.8	490	19.7	417	14.1
Mexico	6650	17.1	5061	9.7	4769	8.1
Nicaragua	392	26.1	414	20.6	424	18.3
Panama	158	13.1	211	12.6	134	7.1
Paraguay	281	15.8	407	16.6	439	15.7
Peru	1575	15.8	2098	16.3	1715	12.0
Puerto Rico	114	4.3	89	2.8	84	2.8
Dominican Republic	495	15.8	591	13.7	600	12.3
Uruguay	163	7.6	159	6.8	175	7.1
Venezuela	1454	15.2	1853	14.4	1789	12.3
Total	29969	12.8	31057	10.7	27998	8.6

Standardised rates per 100,000 women according to the world population model

**Source:** GLOBOCAN (2000, 2008, 2012).

**Table 3.** Cervical cancer in Latin America. Potential years of life lost in 2012.

Countries	Number of deaths	Life expectancy (LE)	Potential years of life lost (PYLL)
Argentina	2127	80	53175
Bolivia	845	69	11830
Brazil	8414	77	185, 108
Chile	734	82	19818
Colombia	1986	78	45678
Costa Rica	116	82	3132
Cuba	569	81	14794
Ecuador	1026	79	24624
El Salvador	388	77	8536
Guatemala	672	75	13440
Haiti	575	65	5750
Honduras	417	76	8757
Mexico	4769	80	119, 225
Nicaragua	424	78	9752
Panama	134	80	3350
Paraguay	439	74	8341
Peru	1715	77	37730
Puerto Rico	84	82	2268
Dominican Republic	600	76	12600
Uruguay	175	80	4375
Venezuela	1789	78	41147
Total	27998		633, 430

**Source:** Deaths, according to GLOBOCAN 2012.

LE: www.bancomundial.org/datos/indicador/SP.DYN%20LE00.FE.IN

PYLL: Estimates according to a mathematical model

**Table 4.** Cervical cancer. Natural history.

Asymptomatic period			Symptomatic period
Susceptible women	Action of risk factors	Preneoplastic stage	Neoplastic stage
Progression	Period from 5 to 20 years	Period from 5 to 15 years	Period from 3 to 10 years
Course of disease	Infection with high-risk HPV and other cofactors	Low- and high-grade intraepithelial lesions	Invasive cancer
Age ranges	Women aged 15 to 40 years	Women aged 20 to 60 years	Women aged 20 to 65 years

**Source:** GLOBOCAN (2000, 2008, 2012) and cancer in the country profiles America 2013.

Cytological cover: Cancer in the Americas: Country Profiles 2013, Washington, DC

**Table 5.** Cervical cancer in Latin America. Correlation between mean mortality rate and cytological cover (2000, 2008, 2012).

Countries	Mortality				Cytological cover
	Standard Rate 2000	Standard Rate 2008	Standard Rate 2012	Mean Standard Rate 2000–2012	
Puerto Rico	4.3	2.8	2.8	3.30	?
Uruguay	7.6	6.8	7.1	7.17	55.20
Chile	10.6	6.6	6	7.73	60.00
Costa Rica	12.1	6.7	4.4	7.73	35.00
Argentina	7.6	7.4	8.4	7.80	60.00
Cuba	10.6	8.9	6.7	8.73	70.00
Brazil	11.6	10.9	7.3	9.93	79.30
Colombia	13.7	10	8	10.57	79.00
Panama	13.1	12.6	7.1	10.93	14.10
Mexico	17.1	9.7	8.1	11.63	70.70
Dominican Republic	15.8	13.7	12.3	13.93	?
Venezuela	15.2	14.4	12.3	13.97	25.00
Peru	15.8	16.3	12	14.70	51.30
Guatemala	16.8	15.2	12.2	14.73	?
El Salvador	15.8	18.2	11.9	15.30	17.80
Ecuador	18.6	13.3	14	15.30	64.30
Paraguay	15.8	16.6	15.7	16.03	?
Honduras	16.8	19.7	14.1	16.87	41.80
Bolivia	22.2	16.7	21	19.97	12.00
Nicaragua	26.1	20.6	18.3	21.67	10.00

Standardised rates per 100,000 women according to the world population model

**Source:** GLOBOCAN (2000, 2008, 2012) and cancer in the America country profiles 2013.a
